# Intermolecular Interactions in the TMEM16A Dimer Controlling Channel Activity

**DOI:** 10.1038/srep38788

**Published:** 2016-12-08

**Authors:** Paolo Scudieri, Ilaria Musante, Ambra Gianotti, Oscar Moran, Luis J. V. Galietta

**Affiliations:** 1 U.O.C. Genetica Medica, Istituto Giannina Gaslini, Genova, Italy; 2 Telethon Institute for Genetics and Medicine (Tigem), Pozzuoli, Italy; 3 Istituto di Biofisica, Consiglio Nazionale delle Ricerche, Genova, Italy

## Abstract

TMEM16A and TMEM16B are plasma membrane proteins with Ca^2+^-dependent Cl^−^ channel function. By replacing the carboxy-terminus of TMEM16A with the equivalent region of TMEM16B, we obtained channels with potentiation of channel activity. Progressive shortening of the chimeric region restricted the “activating domain” to a short sequence close to the last transmembrane domain and led to TMEM16A channels with high activity at very low intracellular Ca^2+^ concentrations. To elucidate the molecular mechanism underlying this effect, we carried out experiments based on double chimeras, Forster resonance energy transfer, and intermolecular cross-linking. We also modeled TMEM16A structure using the *Nectria haematococca* TMEM16 protein as template. Our results indicate that the enhanced activity in chimeric channels is due to altered interaction between the carboxy-terminus and the first intracellular loop in the TMEM16A homo-dimer. Mimicking this perturbation with a small molecule could be the basis for a pharmacological stimulation of TMEM16A-dependent Cl^−^ transport.

TMEM16A and TMEM16B, also known as ANO1 and ANO2, are plasma membrane proteins with Cl^−^ channel function^1^,^2^,^3^. They belong to a family of proteins, called anoctamins, whose other members work as phospholipid scramblases^4^,^5^. TMEM16A is particularly involved in epithelial ion secretion, in nociception, and in smooth muscle mediated contraction of blood vessels, bronchi, and gastrointestinal tract^6^. TMEM16B is instead particularly expressed in neuronal cells (olfactory neurons, photoreceptors, hippocampal neurons) where it is involved in the generation and modulation of electrical signals^7^,^8^,^9^,^10^.

TMEM16A and TMEM16B channels are both regulated by cytosolic Ca^2+^ and membrane potential but with different sensitivities. TMEM16B requires higher (micromolar) concentrations of Ca^2+^ than TMEM16A to be activated^8^,^11^. Furthermore, the kinetics of channel opening and closing are much faster in TMEM16B. Both proteins share regions of high amino acid identity. Other regions, with more divergent sequence, could be responsible for the different biophysical properties. To identify such regions, we previously generated chimerae in which domains of TMEM16A were replaced with equivalent portions of TMEM16B^11^. Unexpectedly, we found that replacement of the entire C-terminal region, localized after the last transmembrane segment, conferred a constitutive activity to the chimeric channel^11^. More precisely, significant membrane currents were observed at very low cytosolic Ca^2+^ concentrations, at which the wild type TMEM16A is totally inactive. Importantly, deletion of the C-terminus in TMEM16A did not generate the same effect. On the other hand, the constitutive activity is not an acquisition of a TMEM16B feature since this channel is even less Ca^2+^-sensitive than TMEM16A. Therefore, such findings suggest that the chimeric C-terminus generates a perturbation of TMEM16A structure that leads to pore opening.

In the present study, we have analyzed in more detail the C-terminus region to identify the precise localization of the “activating domain” and the possible interaction with other regions of TMEM16A. We restricted the critical region to a segment of 14 amino acids and found experimental and theoretical evidence that the C-terminus interacts with the first cytosolic loop of TMEM16A protein.

## Results

**Figure 1:**
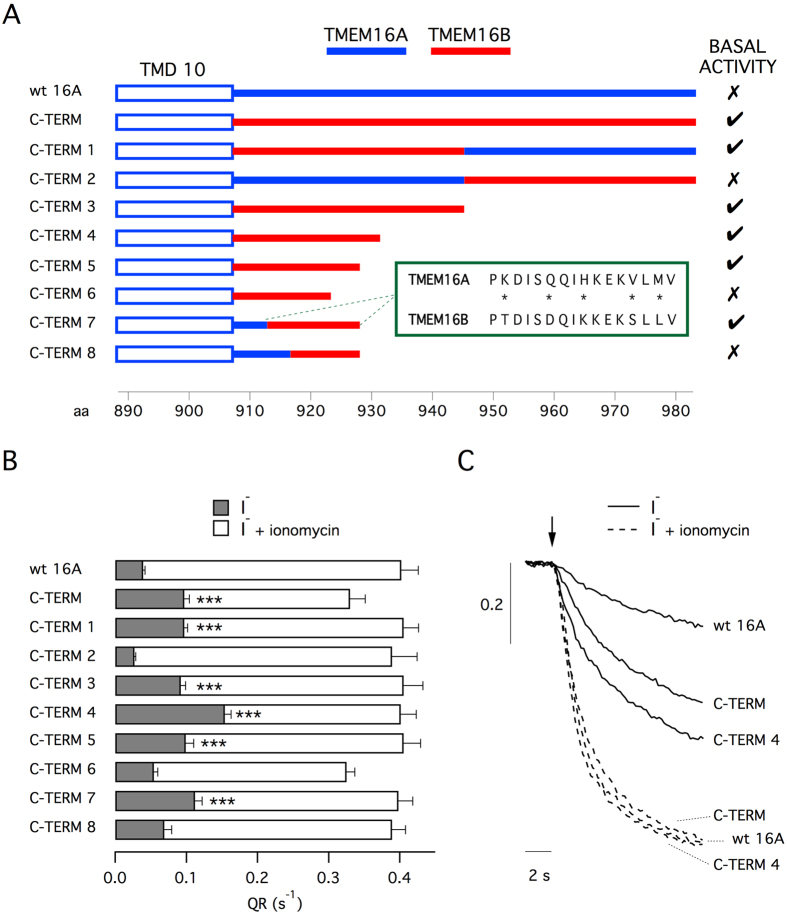
Chimerism of C-terminus potentiates TMEM16A activity. (**A**) Primary structure of the constructs used in the study (wild type and chimeric TMEM16A). Blue and red lines indicate TMEM16A and TMEM16B amino acid sequence, respectively. The precise sequences are reported in Table 1. For each construct, the presence of basal activity (i.e. significant anion transport in the absence of intracellular Ca^2+^ elevation) is indicated. The box reports the alignment between the sequence of TMEM16A and of TMEM16B corresponding to the minimal domain that induces basal activity. Asterisks indicate different amino acid residues. (**B**) Anion transport measured with the HS-YFP assay in HEK-293 cells transfected with the indicated constructs. Data are shown as quenching rate (QR) calculated by fitting the fluorescence decay caused by extracellular I^−^ addition. Gray bars: anion transport without intracellular Ca^2+^ elevation. White bars: anion transport in cells stimulated with 1 μM ionomycin. ***p < 0.001 vs. wild type TMEM16A (n = 12–15 experiments). (**C**) Representative traces of HS-YFP experiments.

In the first part of our study, our goal was to possibly restrict the region of the C-terminus that, when altered, leads to channel activation. The C-terminus of TMEM16A was totally or in part replaced by equivalent regions of TMEM16B and the activity was determined with the halide-sensitive yellow fluorescent protein (HS-YFP) assay^1^,^11^. Measurements of fluorescence decay, reflecting TMEM16A-dependent I^−^ influx, were done with and without stimulation with ionomycin, an ionophore that triggers intracellular Ca^2+^ mobilization. Figure 1A depicts the different versions of TMEM16A that were used for experiments. Besides replacing segments of TMEM16A with those of TMEM16B, we also truncated the C-terminus at different levels. Figure 1B shows functional data obtained with the various TMEM16A constructs transfected in HEK-293 cells. Activity of the different constructs is presented as quenching rate (QR) calculated from the fitting of fluorescence decay traces (Fig. 1C). For wild type TMEM16A, the basal activity measured in the absence of ionomycin was less than 10% of that elicited by stimulation (compare gray and white bars). This percentage increased to 30% when the C-terminus was totally replaced with the TMEM16B sequence (C-TERM construct). This result confirms previous observations^11^. We then tested two constructs in which only the first half (C-TERM1) or the second half (C-TERM2) of the C-terminus was replaced. Importantly, only modification of the first part of the C-terminus increased the basal activity, thus restricting the localization of the “activating domain”. Subsequent constructs, from C-TERM3 to C-TERM6, were done by replacing the TMEM16A C-terminus with progressively truncated versions of the TMEM16B C-terminus. The experiments showed that C-TERM4, having 25 replaced amino acids, had the maximal basal activity (40%). Shorter versions were less active. C-TERM6, having only 16 replaced amino acids, was comparable to wild type TMEM16A. We also studied two additional constructs, C-TERM7 and C-TERM8, in which the amino acids closer to the transmembrane domain were not replaced. Only C-TERM7 kept the basal activity. Importantly, the white bars in Fig. 1B, reflecting activity when the channels were maximally stimulated with ionomycin, are of similar size for most of the TMEM16A constructs. Such results suggest similar transfection/expression efficiency for the different constructs in agreement with previous results with this assay^1^,^11^. Therefore, the different values of QR observed in the absence of ionomycin are probably due to intrinsic differences in channel gating.

Summarizing, of the 76 amino acids that compose the C-terminus of TMEM16A, replacement of the residues 913-926 is sufficient to generate significant activity in the absence of intracellular Ca^2+^ elevation. It is important to note that in this segment, only 5 residues are different between TMEM16A and TMEM16B (Fig. 1A). To clarify the contribution of these amino acid residues, we progressively mutagenized each residue and determined the effect on TMEM16A activity (Supplementary Fig. S1A–C). We started with the replacement of histidine 920 with the lysine of TMEM16B in the center of the domain (mutation H920K, labeled as mutant 16A-K in Supplementary Fig. S1B,C). Activity was determined with the HS-YFP assay without ionomycin stimulation. The assay showed no increase in basal activity for 16A-K with respect to wild type TMEM16A (Supplementary Fig. S1B,C). No significant change was also observed for the double mutant H920K/Q917D (16A-DK). Instead, introduction of a third mutation, V924S (16A-DKS), significantly increased basal activity (Supplementary Fig. S1B,C). The triple mutant 16A-DKS was used to introduce a fourth mutation, either K913T or M926L (16A-TDKS and 16A-DKSL, respectively). Such changes caused little change to activity compared to the triple mutant. Instead, mutagenesis of all five residues (16A-TDKSL) significantly increased basal activity over triple or quadruple mutants (Supplementary Fig. S1B,C). Summarizing, the properties of the “activating domain” from TMEM16B are not due to a single position but distributed among more than one amino acid residue.

**Figure 2:**
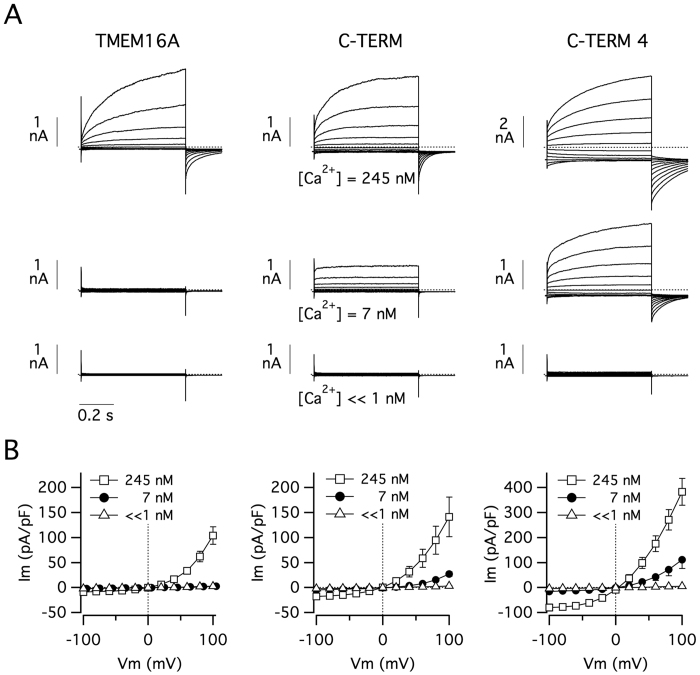
Altered membrane currents in TMEM16A proteins with chimeric C-termini. (**A**) Representative membrane currents in HEK-293 cells expressing wild type TMEM16A or C-TERM/C-TERM4 mutants. Each panel reports superimposed currents elicited at different membrane voltages (−100 to +100 mV range) and at the indicated intracellular free Ca^2+^ concentrations. (**B**) Current-voltage relationships summarizing the results of experiments as those shown in (**A**). Each point is the mean ± SEM of 9–12 experiments.

The effect of C-terminus modification was also evaluated with the patch-clamp technique in the whole-cell configuration (Fig. 2). For such experiments, we transfected HEK-293 cells with wild type TMEM16A, C-TERM, or C-TERM4. Membrane currents were measured with three pipette (intracellular) solutions having different free Ca^2+^ concentrations. With 245 nM, all three constructs showed large membrane currents with the typical voltage-dependence, i.e. activation and deactivation at depolarizing and hyperpolarizing membrane potentials, respectively. However, it should be noted that the currents generated by C-TERM4 were more than two-fold larger (Fig. 2A; note the different scale bar). As expected, with 7 nM, wild type TMEM16A was totally inactive. At the same Ca^2+^ concentration, C-TERM and C-TERM4 mutants showed significant membrane currents although with different properties. As described previously for this low level of cytosolic Ca^2+ ^11, C-TERM expression generated currents of modest amplitude, with strong outward rectification and lack of time-dependent activation at positive membrane potentials (Fig. 2A). Negligible tail currents were obtained when the membrane potential was returned to the holding value (−60 mV) from a depolarizing step (e.g. +100 mV). Instead, with 7 nM Ca^2+^, the currents associated with C-TERM4 were comparable, in terms of amplitude and shape, to those observed for wild type TMEM16A at 245 nM Ca^2+^ (Fig. 2A). When a solution with very low Ca^2+^ (<1 nM) was used, none of the constructs showed activity. As evident from the current-voltage relationships of Fig. 2B, C-TERM4 shows significant inward currents at negative membrane potentials when the intracellular free Ca^2+^ concentration is as low as 7 nM.

**Figure 3:**
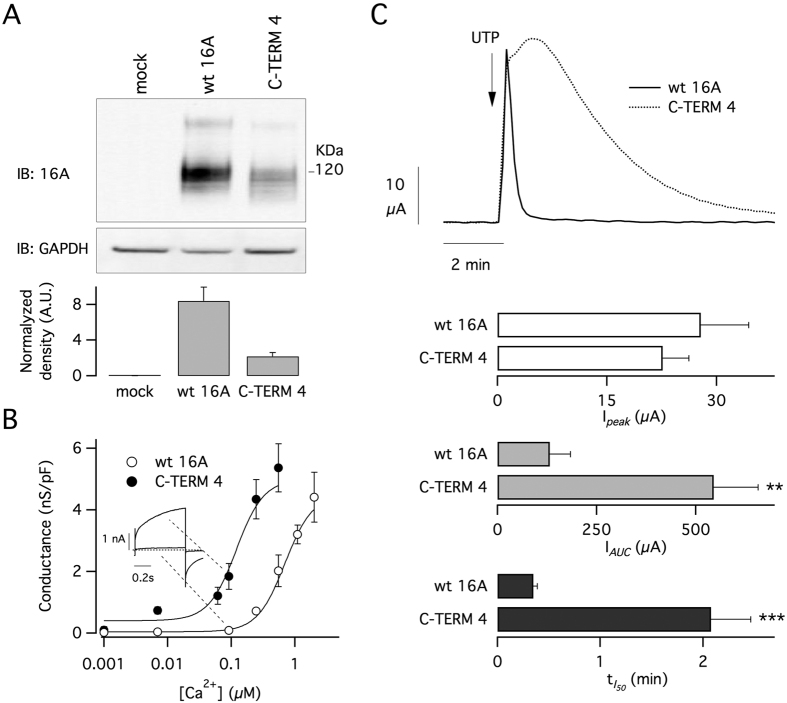
Altered Ca^2+^-sensitivity of chimeric TMEM16A channels. (**A**) Analysis of TMEM16A expression in FRT cells with stable expression of the wild type protein or chimeric C-TERM4 construct. Top: representative Western blot experiment. Bottom: densitometric analysis from 3 experiments. (**B**) Analysis of Ca^2+^-sensitivity of TMEM16A channels in FRT cells. The graph shows the membrane conductance measured by whole-cell patch-clamp experiments at the indicated intracellular free Ca^2+^ concentrations. Membrane conductance was measured from tail currents as described in Methods. (**C**) Results from transepithelial current recordings in FRT cells. Top: representative recordings of responses to apical UTP (100 μM) application. Bottom: bar graphs reporting the properties of currents elicited in cells expressing wild type protein or C-TERM4 (peak, the area under the curve, and time required for the current to decay to 50% of maximal value. **p < 0.01; ***p < 0.001 vs. wild type protein (n = 9 experiments).

To further confirm these results, we expressed C-TERM4 construct in FRT cells and compared its properties with those of wild type TMEM16A. As shown in Fig. 3A, we first investigated the protein expression levels by Western blot using a primary antibody against TMEM16A. The FRT clone with C-TERM4 showed a nearly four-fold lower expression compared to the cells with the wild type protein. Despite this lower expression, C-TERM4 cells had large membrane currents as indicated by patch-clamp experiments done at different intracellular Ca^2+^ concentrations (Fig. 3B). In particular, the relationship between membrane conductance, calculated from tail currents (see methods), and Ca^2+^ concentration appeared to be shifted by an order of magnitude to the left with respect to wild type TMEM16A (Fig. 3B).

FRT cells form electrically tight epithelia when seeded on porous membranes (Snawpell inserts). Therefore, FRT cells expressing wild type TMEM16A and C-TERM4 were also studied in short-circuit current recordings in which channel activity results in transepithelial currents. Cells were stimulated with apical application of UTP (100 μM) to induce intracellular Ca^2+^ mobilization. Figure 3C shows that the two types of cells responded differently to stimulation. Although the UTP-activated current reached similar maximal values in both types of cells, it lasted longer in cells with C-TERM4 as indicated by two parameters: the area under the curve (AUC) and the time required by the current to decay to half of its maximal value (Fig. 3C).

**Figure 4:**
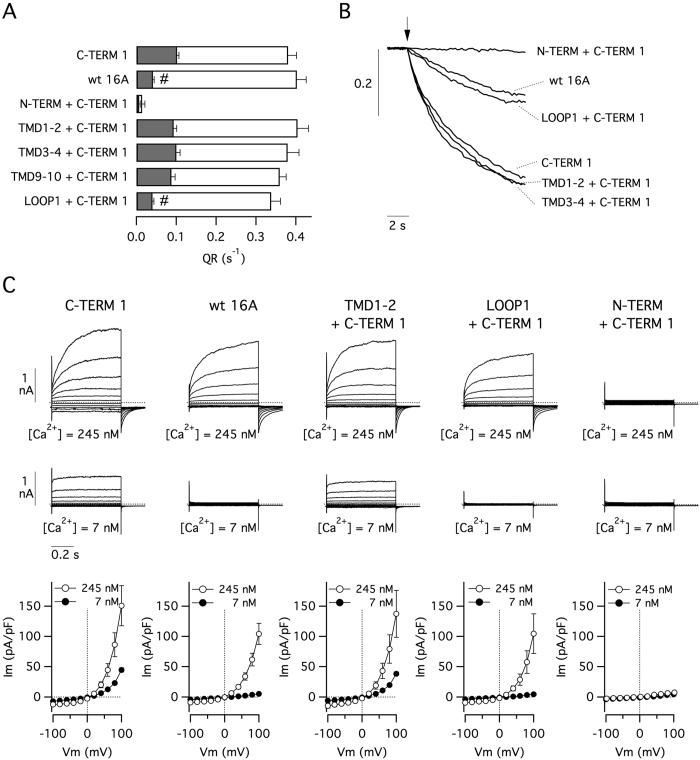
Channel activity in double chimeric TMEM16A proteins. (**A**) Results from HS-YFP assay carried out on HEK-293 cells expressing the indicated constructs. Gray bars: anion transport in the absence of intracellular Ca^2+^ elevation. White bars: anion transport after stimulation with ionomycin (1 μM). ^#^p < 0.05 vs. C-TERM1 (n = 6–9 experiments). (**B**) Representative traces from HS-YFP experiments. (**C**) Representative whole-cell membrane currents and current-voltage relationships from 6–9 experiments with the indicated TMEM16A constructs. Intracellular free Ca^2+^ concentration was 7 or 245 nM.

We hypothesized that the C-terminus of TMEM16A normally interacts with another domain of the protein and that modification of C-terminus sequence perturbs this interaction. This could be the mechanism explaining the increase in activity at low Ca^2+^ concentrations occurring with C-TERM mutants. We also hypothesized that generating TMEM16A double chimerae, in which both the C-terminus and its interacting domain derive from TMEM16B, could lead to restoration of the normal domain-domain interaction and activity. Figure 4A,B shows data obtained with the HS-YFP assay. HEK-293 cells were transfected with constructs having both the C-TERM1 modification and another domain also deriving from TMEM16B. We found that basal activity associated with C-TERM1 disappeared only when the first intracellular loop (LOOP1) was also modified. Interestingly, channels became totally inactive when the double replacement affected both the carboxy- and the amino-terminus. The results obtained with the HS-YFP assay were confirmed in patch-clamp experiments. The currents measured with 7 nM Ca^2+^ were absent in the double chimaera LOOP1+C-TERM1 (Fig. 4C).

**Figure 5:**
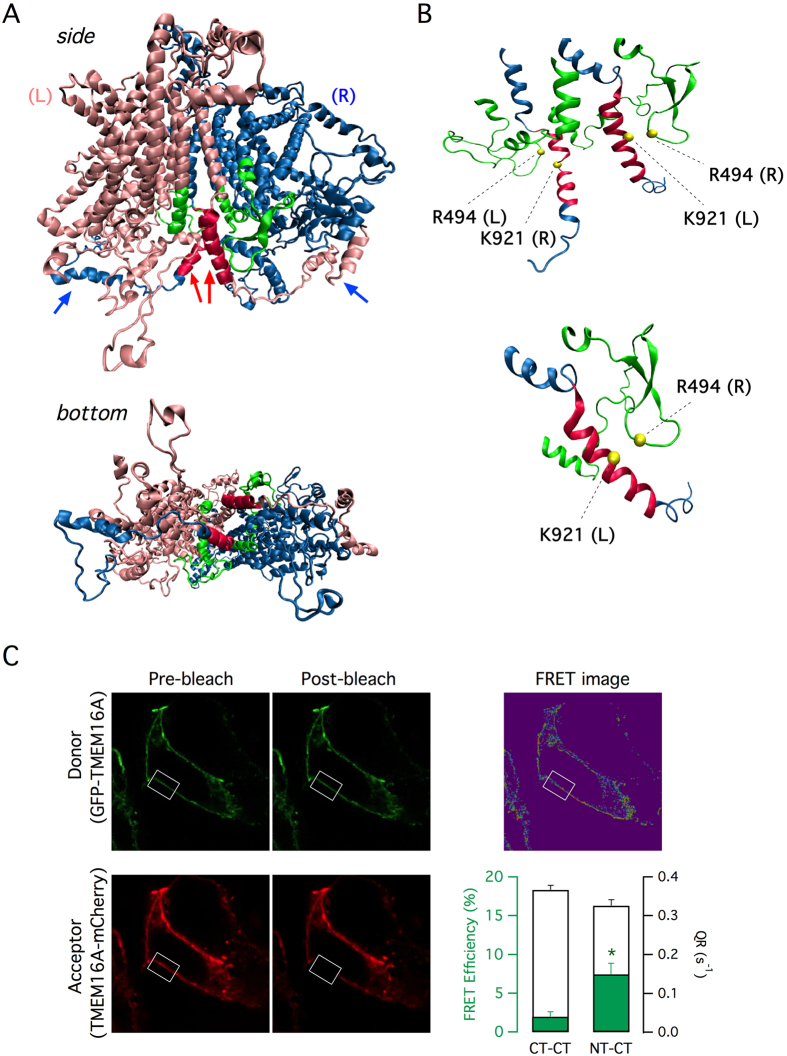
Homology model for TMEM16A protein. **(A**) Side and bottom view of the TMEM16A homodimer. The left and right monomers are colored in magenta and blue respectively. C-termini from each side first meet at the center of the dimer (colored in red and indicated by red arrows). The C-termini then extend to the other side of the dimer and take contact with the N-terminus of the opposite subunit (blue arrows). **(B**) Close view of the two C-termini and the two first intracellular loops. The position of the residues R494 and K921 (subsequently mutagenized to cysteines in Fig. 6) are indicated. (**C**) Results from acceptor photobleaching FRET experiments. Representative images show fluorescence of GFP and mCherry fused to the N-terminus and C-terminus of TMEM16A, respectively (NT-CT configuration). Images were taken before and after bleaching the acceptor (mCherry) in the area indicated by the rectangle. The green bars in the graph show the calculated FRET in experiments with NT-CT or CT-CT configuration (GFP and mCherry both fused to the C-terminus). *p < 0.05 vs. CT-CT, n = 15 experiments. The white bars in the graph show QR from HS-YFP experiments; both NT-CT and CT-CT configurations generate functional TMEM16A channels.

Recently, the tridimensional structure of a TMEM16 protein was determined by X-ray crystallography^12^. This structure, as well as previous biochemical studies on TMEM16A^13^,^14^, indicated that anoctamins form homodimers. We used this information to model the structure of TMEM16A. Figure 5A shows an hypothetical view of TMEM16A from the side (parallel to the membrane) and the bottom (from the intracellular side). Each subunit of the dimer contains ten transmembrane domains and cytosolic N- and C-termini. The carboxy-terminus of each monomer is wrapped around the cytosolic face of the adjacent subunit. In particular, the carboxy-terminal domain is composed of three α-helices. The most proximal helix (Fig. 5A, red arrows) appears to be close to the first intracellular loop of the ipsilateral and contralateral subunits. Instead, the other two α-helices, as already shown for nhTMEM16^12^, take contact with the N-terminal domain of the contralateral subunit (Fig. 5A, blue arrows). A detailed view of the region of proximity between the C-terminus and the first intracellular loop is shown in Fig. 5B. In particular, the figure shows the position of residues (R494 in the first intracellular loop, K921 in the C-terminus) that were subsequently used for mutagenesis. It is important to note that the α-helix of the C-terminus that is close to the first intracellular loop contains the aminoacidic sequence corresponding to the “activating domain” of TMEM16B.

To gain insights into the possible interaction between the C-terminus and the first intracellular loop of TMEM16A protein, we carried out molecular dynamics simulations on the homology model of wild type TMEM16A and of C-TERM4. We first examined the inter-residues average distance between the first α-helix of the C-terminus and first intracellular loop of the ipsilateral and contralateral subunits (Supplementary Figs S2 and S3). For both the wild type TMEM16A and the C-TERM4 mutant, this analysis identified a series of possible contact sites, particularly between the first intracellular loop and the C-terminus of the contralateral subunits (Supplementary Figs S2A and S3A). As second parameter, we analyzed the variance of the inter-residues distance. Molecular dynamics simulations revealed for the wild type TMEM16A very small distance fluctuations between the C-terminus and the first intracellular loop (Supplementary Figs S2B and S3B). Instead, C-TERM4 mutant showed larger distance fluctuations, particularly between the C-terminus and residues 492-498 in the first intracellular loop of the ipsilateral subunit (Supplementary Figs S2B and S3B).

To validate the theoretical 3D model of TMEM16A, we used two approaches. The first one was based on FRET experiments. From the model, the distance between the C-terminus of one monomer and the N-terminus of the other monomer (4 nm) is smaller than the distance between the two C-termini. Therefore, we coexpressed in HEK-293 cells pairs of TMEM16A proteins fused to GFP and mCherry. In one pair, TMEM16A-GFP and TMEM16A-mCherry, the two fluorescent proteins were fused separately to the C-terminus of TMEM16A. In the second pair, GFP-TMEM16A and TMEM16A-mCherry, one of the two TMEM16A proteins had GFP fused to the N-terminus. Using the acceptor photobleaching method, we compared the FRET efficiency for the two pairs of fluorescent TMEM16A proteins (Fig. 5C, green bars). FRET efficiency between TMEM16A-GFP and TMEM16A-mCherry (CT-CT configuration) was 1.97 ± 0.61%. Instead, The FRET efficiency between GFP-TMEM16A and TMEM16A-mCherry (NT-CT configuration) was significantly higher, 7.44 ± 1.43%. Such results are in agreement with the predicted distances between the N- and C-termini in the TMEM16A dimer. We considered the possibility that lack of significant FRET for the CT-CT configuration could actually arise from impairment in TMEM16A dimer assembly, due to addition of fluorescent proteins at both C-termini. Therefore, we measured anion transport for CT-CT and NT-CT. Our results showed similar levels of activity thus indicating that CT-CT configurations generates functional channels (Fig. 5C, white bars).

The second approach to validate the 3D model was based on inter-molecular cross-linking with a bifunctional cysteine reagent. For this purpose, we generated a version of TMEM16A devoid of cysteines in the cytosolic portion (16A-cys(i)-less). An entire cysteine-free TMEM16A could not be generated since removal of extracellular cysteines leads to total loss of activity^15^. In the 16A-cys(i)-less sequence, we introduced a cysteine at position 494 (R494C), a cysteine at position 921 (K921C), or two cysteines at both positions (R494C/K921C). According to the TMEM16A dimer model, the distances between the two arginines 494, between the two lysines 921, and between arginine 494 and lysine 921 of the opposite subunits are 29 Å, 23.7 Å, and 10.8 Å, respectively. We reasoned that using bifunctional cross-linkers reacting with cysteines, we could induce the formation of covalently linked TMEM16A dimers resulting in a shift of electrophoretic mobility. As a control, we also introduced a cysteine at position 285 (S285C). The predicted distance between the two C285 residues are 120 Å.

**Figure 6:**
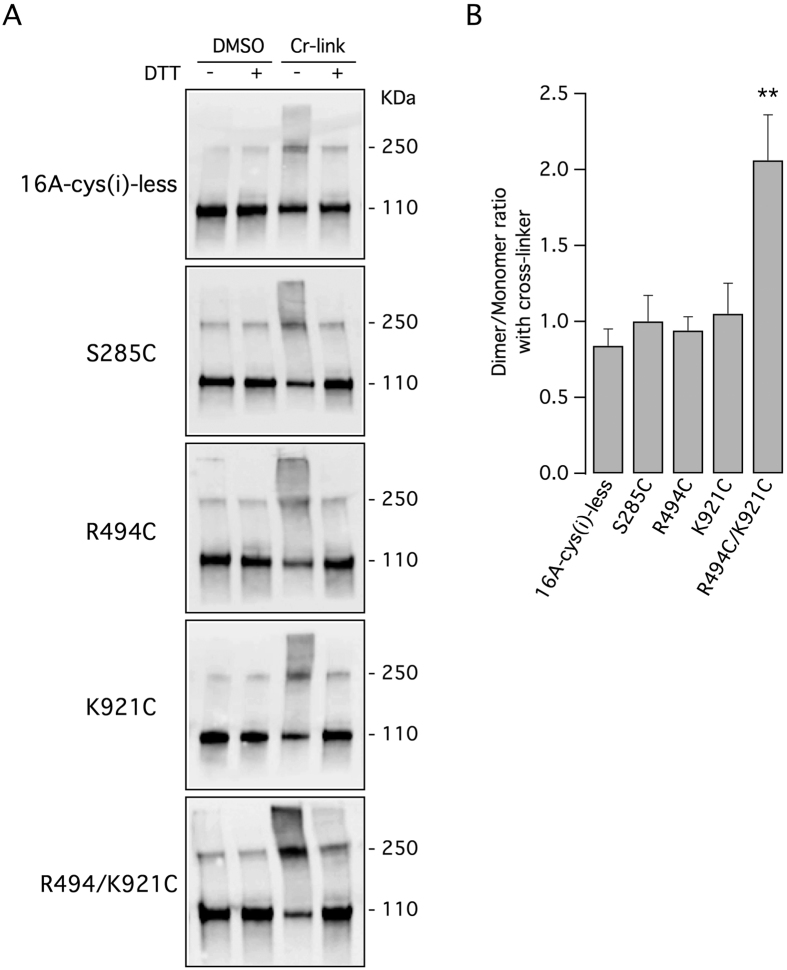
Intermolecular cross-linking of TMEM16A protein. (**A**) Electrophoretic mobility of TMEM16A protein in the presence or absence of a bifunctional cysteine-reacting molecule, with and without the reducing molecule DTT. Experiments were done on lysates from cells expressing a version of TMEM16A completely devoid of intracellular cysteines (16A-cys(i)-less) or with cysteines introduced at the indicated positions. (**B**) The bar graph shows the dimer/monomer ratio deriving from densitometric analysis of lanes corresponding to cells treated with the cross-linker. **p < 0.01 vs. the cysteine-less construct, n = 5 experiments.

[Table t1]In the absence of cross-linker, the 16A-cys(i)-less construct mostly migrates as a monomer (110 kDa; Fig. 6A, top panel). There is a faint band corresponding to the dimer (250 kDa) that suggests, as previously reported^11^, the presence of detergent-resistant non-covalent bonds between the two TMEM16A molecules in the dimer. Addition of the cross-linker resulted in increased levels of TMEM16A dimer, an effect that could be reversed by the DTT reducing agent (Fig. 6A, top panel). The effect of the cross-linker on this TMEM16A construct can be attributed to extracellular cysteines. When the cross-linker was added to cells expressing 16A-cys(i)-less with single intracellular cysteines (S285C, R494C, and K921C) the results were not significantly different from those obtained without intracellular cysteines (Fig. 6A,B). In contrast, with the double mutant R494C/K921C, we observed a marked increase in the intensity of the band corresponding to the dimer (Fig. 6A, bottom panel). This effect was reversed by DTT. The dimer/monomer ratio for the double mutant was significantly higher than those of the other constructs (Fig. 6B).

## Discussion

The molecular mechanisms that control the activity of TMEM16A protein is quite complex and not fully understood. Here, we attempted to identify protein regions that determine the channel gating. Activation by Ca^2+^ appears to require the binding to specific residues localized in transmembrane domains 6, 7 and 8^15^,^16^. Ca^2+^ binding to these sites probably induce large conformational changes that involve other TMEM16A domains. In this respect, the first intracellular loop of TMEM16A was found to control the sensitivity of the channel to Ca^2+^ and/or to membrane potential^17^,^18^. In a previous study, by generating chimeric proteins in which regions of TMEM16A were replaced by equivalent regions of TMEM16B, we obtained channels with altered properties^11^. In particular, we found that the proteins with chimeric C-terminus showed increased activity at low intracellular Ca^2+^ concentrations. Importantly, simple deletion of the C-terminus did not reproduce the same effect^11^. This finding suggested that modification of the C-terminus causes a gain of function probably by altered interaction with another region of the protein.

In the present study, we have restricted the part of the C-terminus that is responsible for altered channel activity. In particular, we have identified a minimal region of 14 amino acids close to the last transmembrane domain that appears to affect the sensitivity of the channel to very low Ca^2+^ concentrations. It is interesting to note that in this region only five amino acids are different between TMEM16A and TMEM16B. In intact cells (HS-YFP and transepithelial electrical recordings), this change in behavior resulted in channels with increasing activity. Importantly, Ca^2+^-dependence was not abolished since complete removal of nominal Ca^2+^ fully inactivated the channels.

To identify the domain that interacts with the C-terminus, we generated double chimeras. These experiments evidenced the intracellular loop as the possible region of interaction. Indeed, only the double chimaeras that had both the first intracellular loop and the C-terminus derived from TMEM16B had normal activity. The link between these two domains also arise by modeling TMEM16A with the nhTMEM16 structure as template^12^. According to the homology model, each C-terminus crosses the gap at the center of the TMEM16A dimer and ends on the other side taking contact with N-terminus of the opposite monomer. To support this model, we carried out two types of experiments. By FRET, we found evidence that the last part of the C-terminus of one TMEM16A monomer is indeed close to the N-terminus of the other monomer. Our results are therefore in agreement with the structure postulated for nhTMEM16^12^, a distant fungal paralog of TMEM16A, suggesting a common overall spatial organization of TMEM16 proteins working as channels and scramblases^19^. By intermolecular cross-linking, we found results consistent with a close distance between the most proximal part of each C-terminus and the first intracellular loop of the opposite subunit. Indeed, a bifunctional cross-linking reagent significantly increased the dimer/monomer ratio only when two cysteines were introduced at the closest predicted distance. Therefore, the C-termini and the first intracellular loops meet at the center of TMEM16A dimer forming a complex that may be important for channel structure and function. As also evidenced by molecular dynamics simulation, modification of the C-terminus sequence can perturb this complex leading to potentiation of channel activity. Importantly, mimicking this perturbation with small molecules could be the basis for a pharmacological approach to stimulate TMEM16A-dependent ion transport. Activation of TMEM16A by drugs could be useful to stimulate epithelial anion secretion in the airways, an effect potentially beneficial for cystic fibrosis and other chronic obstructive respiratory diseases^20^.

## Methods

### Cell culture and transfection

HEK-293 and HEK-293 MSR cells (Life Technologies) were cultured in DMEM/Ham’s F12 supplemented with 10% fetal bovine serum, 2 mM L-glutamine, 100 U/ml penicillin, and 100 μg/ml streptomycin.

For the functional assay based on the halide-sensitive yellow fluorescent protein (HS-YFP)^1^,^21^, HEK-293 MSR cells were seeded in 96-well microplates (30,000 cells/well) in 150 μl of antibiotic-free culture medium. After 24 hours, cells were co-transfected with plasmids carrying the coding sequence for TMEM16A (wild type and chimeric) and the HS-YFP. For each well, 0.2 μg of total plasmid DNA and 0.5 μl of Lipofectamine 2000 (Invitrogen) were first pre-mixed in 50 μl of OPTIMEM (Invitrogen) to generate transfection complexes (60 minutes at room temperature), and then added to the cells. After 24 hour, the complexes were removed by replacement with fresh culture medium. The YFP-based functional assay was performed after further 24 hours.

For patch-clamp experiments, 500,000 HEK-293 cells in 1.5 ml of antibiotic-free culture medium were mixed with 0.5 ml of OPTIMEM containing 2 μg of plasmid DNA and 5 μl of Lipofectamine 2000 (previously incubated for 60 minutes at room temperature to allow formation of lipid/DNA complexes). Cell suspension was then plated as 6–7 drops in 35 mm Petri dishes. After 6 hours, 2 ml of culture medium without antibiotics were added to each Petri dish. After 24 hours, the complexes were removed by replacement with fresh culture medium. Patch-clamp experiments were carried out 2 days after transfection.

Fischer rat thyroid (FRT) cells were cultured in Coon’s modified Ham’s F-12 medium supplemented with 10% fetal calf serum, 2 mM L-glutamine, 100 U/ml penicillin, and 100 μg/ml streptomycin. For generation of stably-transfected clones, FRT cells were plated in six-well microplates (300,000 cells/well). After 24 hours, cells were transfected with the plasmids coding for wild type TMEM16A or C-TERM 4 (2 μg of plasmid and 5 μl of Lipofectamine 2000 per well). After 24 hours, cells were selected for 3–4 days with G418 1 mg/ml in the culture medium. After this initial phase, cells were detached by trypsinization and seeded in 96-well microplates at a density of approximately one cell per well. Selection in G418 was continued for further 2–3 weeks until single cell colonies were identified by visual inspection. Cells from each well were trypsinized and further expanded. Clones expressing wild type or mutant TMEM16A were identified by immunofluorescence^22^.

### HS-YFP assay

Transiently transfected HEK-293 MSR cells were rinsed two times with 200 μl of PBS (137 mM NaCl, 2.7 mM KCl, 8.1 mM Na_2_HPO_4_, 1.5 mM KH_2_PO_4_, 1 mM CaCl_2_, and 0.5 mM MgCl_2_) and incubated for 30 minutes with 60 μl of the same solution. After incubation, 96-well microplates were transferred to a microplate reader (FluoStar Galaxy; BMG Labtech) equipped with high-quality excitation (ET500/20X: 500 ± 10 nM) and emission (ET535/30M: 535 ± 15 nM) filters for YFP (Chroma Technology Corp., Brattleboro, VT). For each well, cell fluorescence was continuously measured for 2 seconds before and 12 seconds after injection of 165 μl of a modified PBS containing 137 mM KI instead of NaCl (final I^−^ concentration in the well: 100 mM). Where indicated, this solution also contained ionomycin (1 μM) as a Ca^2+^-elevating agent. After background subtraction, cell fluorescence recordings were normalized for the initial value measured before addition of I^−^ in each well. The signal decay caused by HS-YFP fluorescence quenching was fitted with a double exponential function to derive the maximal quenching rate (QR) that corresponds to initial influx of I^−^ into the cells.

### Patch-clamp recordings

Whole-cell membrane currents were recorded in HEK-293 transiently-transfected cells and in FRT stably-transfected cells. The extracellular solution had the following composition: 150 mM NaCl, 1 mM CaCl_2_, 1 mM MgCl_2_, 10 mM glucose, 10 mM mannitol, 10 mM Na-HEPES (pH = 7.4). The pipette (intracellular) solution contained: 130 mM CsCl, 10 mM EGTA, 1 mM MgCl_2_, 10 mM HEPES, 1 mM ATP (pH = 7.4) plus CaCl_2_ to obtain the desired free Ca^2+^ concentration: 1 mM for 7 nM, 5 mM for 61 nM, 6 mM for 92 nM, 8 mM for 245 nM, 9 mM for 551 nM, 9.5 mM for 1.1 μM, 9.71 mM for 2 μM (calculated with the Ca/Mg/ATP/EGTA Calculator v2.2b available at http://www.stanford.edu/~cpatton/maxc.html). We also used a solution without nominal CaCl_2_ and containing 10 mM EGTA (indicated in Fig. 2 as ≪1 nM). During experiments, the membrane capacitance and series resistance were analogically compensated using the circuitry provided by the EPC7 patch-clamp amplifier. The usual stimulation protocol to generate current-voltage relationships consisted in 600 ms-long voltage steps from −100 to +100 mV in 20 mV increments starting from a holding potential of −60 mV. The waiting time between steps was 4 s. Membrane conductance was calculated from the tail current measured at −60 mV following a pulse at +100 mV. Membrane currents were filtered at 1 kHz and digitized at 5 kHz with an ITC-16 (Instrutech) AD/DA converter. Data were analyzed using the Igor software (Wavemetrics) supplemented by custom software. Data are reported as representative traces or mean ± standard error of the mean.

### Generation of chimeric and truncated constructs

The wild type TMEM16A(*abc*) coding sequence was cloned into the pcDNA 3.1 plasmid as described previously^1^.

To generate chimeric and double-chimeric constructs we used a strategy based on gene synthesis provided by GeneCust (Dudelange, Luxemburg). For this purpose, we introduced a BspEI restriction enzyme site by mutagenizing a single nucleotide at position 1097 of the TMEM16A(*abc*) coding sequence. This mutation did not change the amino acid sequence, nor the extent of protein expression and activity as shown by functional measurements^11^. By using the BspEI site, an endogenous EcoRI site localized at position 1836, and KpnI and XhoI flanking the cloning site, we could split the TMEM16A coding sequence in three parts of grossly similar length. Accordingly, each chimera was generated by designing the synthesis of a DNA fragment flanked by a pair of restriction sites and containing the desired combination of TMEM16A and TMEM16B sequence. This fragment was cut and pasted into the wild type TMEM16A sequence using the proper restriction enzymes.

Generation of truncated constructs was done with the QuickChange XL site-directed mutagenesis kit (Stratagene) by introducing a stop codon at the following positions of the C-TERM 1 construct: 945 for C-TERM3, 932 for C-TERM4, 928 for C-TERM5, 923 for C-TERM6. C-TERM7 and C-TERM8 were generated by introducing, respectively, 1 and 2 missense mutations in the C-TERM 5 construct. All plasmids were checked by DNA sequencing.

### Transepithelial Cl^−^ current measurements

FRT cells with stable expression of TMEM16A or C-TERM 4 were plated on Snapwell inserts 3801 (500,000 cells per insert; Corning Life Sciences). Experiments were performed 7 days after plating by mounting the Snapwell inserts in a self-contained Ussing chamber system (vertical diffusion chamber). Transepithelial currents were measured using a transepithelial chloride gradient. Accordingly, the basolateral solution contained 130 mM NaCl, whereas the apical solution contained 65 mM NaCl and 65 mM sodium gluconate. The two solutions had also 2.7 mM KCl, 1.5 mM KH_2_PO_4_, 1 mM CaCl_2_ (2 mM for apical), 0.5 mM MgCl_2_, 10 mM Na-HEPES (pH 7.5), and 10 mM glucose. During experiments, solutions in both chambers were continuously bubbled with air. The hemichambers were connected to DVC-1000 voltage clamp (World Precision Instruments, Inc., Sarasota, FL) via Ag/AgCl electrodes and agar bridges (1 M KCl in 1% agar). Transepithelial currents were digitized using a PowerLab 4/25 data acquisition system (ADInstruments) and stored on a personal computer. All measurements were done at 37 °C.

### Homology modelling of wild-type and mutant TMEM16A channels

The molecular structure of wild type TMEM16A and the structure of the mutant C-TERM 4 were modelled using the crystal structure of the lipid scramblase nhTMEM16 (PDB 4WIS). For each construct, the program Yasara^23^,^24^ was used to generate 5 models with alternative aligments between the template and the target sequences. The best parts of the 5 models were combined to obtain a hybrid model, to increase the accuracy beyond each of the contributors, obtaining an average Z-score of -1.939.

### Energy minimisation and molecular dynamics

Energy minimisation and molecular dynamics (MD) were performed with the program NAMD^25^ using the Charmm27 force field. A first energy minimisation was performed to remove the gross molecule errors. Subsequently, the model was inserted into a lipid membrane and hydrated using the routines available in VMD^26^. A 1-palmitoyl-2-oleoyl-sn-glycero-3-phosphocholine (POPC) bilayer of 150 × 150 Å was used while the two sides of the bilayer were solvated with standard TIP3 water molecules. Sodium and chloride ions corresponding to a concentration of 150 mM were included in the water phase to neutralize the charges of the system. The system was extensively minimised by restraining protein heavy atoms and allowing water molecules to equilibrate the protein–solvent interactions. The system was heated to 300 K in a stepwise manner at a constant pressure of 1 atm. A MD simulation at 300 K was run for 10 ns to equilibrate the system. The calculation interval for the equations of motion was 2 fs. Long-range electrostatic interactions were calculated using the Ewald approximation using periodic boxes. The box side was at least at 20 Å apart from the protein atoms. The SHAKE^27^ procedure was employed to constrain all bonds connecting hydrogen atoms. Non-bonded energy terms were calculated with smoothing cut-off functions to both the electrostatics and van der Waals forces, starting at 10 Å, to a complete cut-off at 12 Å. The Ewald sum was computed using the Particle-Mesh Ewald (PME) method as implemented in the NAMD program^25^. All analyses were performed using Yasara, VMD and self-written software.

### FRET experiments

HEK-293 cells, seeded on 8-well μ-slide (80826, IBIDI) were co-transfected with eGFP-TMEM16A and TMEM16A-mCherry or TMEM16A-eGFP and TMEM16A-mCherry. FRET was performed using a TCS-SP8 confocal microscope (Leica Microsystems) with a 63x oil immersion objective. The donor (eGFP) was excited at 488 nM and emission collected from 495 to 540 nM, and the acceptor (mCherry) was excited at 561 nM and emission collected from 580 to 650 nM. FRET was measured by the acceptor photobleacing method with the FRET wizard in Leica Application Suite Advanced Fluorescence (LAS AF) software.

### Cysteine cross-linking and Western blot detection of TMEM16A protein

A version of TMEM16A in which all cysteines in the cytosolic portion were replaced with serines (16A-cys(i)-less) was generated by gene synthesis (GeneCust) and cloned into pcDNA3.1 plasmid.

HEK-293 cells seeded on 6-well plates (300.000 cells/well) were transfected with 16A-cys(i)-less or mutants containing 1 or 2 cysteines in the cytosolic portion (S285C, R494C, K921C, R494C/K921C). 48 hours after transfection, cells were washed with PBS, harvested and resuspended in 50 μl of PBS. Then, 10 μl of cell suspension was mixed with 20 μl of PBS containing 300 μM cross-linker (1,5-pentanediyl bismethanethiosulfonate; Toronto Research Biochemicals) and incubated at room temperature. After 15 minutes, the cross-linking reaction was reversed by reduction with 33 mM DTT or directly stopped with Laemmli sample buffer (3% SDS, 10% glycerol, 62,5 mMTris-HCl pH 6,8, 1 mM EDTA). Six microliters of the samples were separeted onto Mini-Protean TGX precast Gels 4-15% (Biorad) and transferred to PVDF membrane (Biorad) for Western blotting with Trans-Blot Turbo system (Biorad). TMEM16A protein was immunodetected by a rabbit monoclonal antibody (SP31, Abcam) diluted 1:10000 in 5% skimmed-milk in TBS-T. After washings and incubation with peroxidase-coupled secondary antibody, protein bands were detected using the Super Signal West Femto Maximum Sensitivity Substrat (Thermo Fisher Scientific Inc). Direct recording of the chemiluminescence was performed using the Molecular Imager ChemiDoc XRS System (Biorad).

## Additional Information

**How to cite this article**: Scudieri, P. *et al*. Intermolecular Interactions in the TMEM16A Dimer Controlling Channel Activity. *Sci. Rep.* **6**, 38788; doi: 10.1038/srep38788 (2016).

**Publisher's note:** Springer Nature remains neutral with regard to jurisdictional claims in published maps and institutional affiliations.

## Supplementary Material

Supplementary Information

## Figures and Tables

**Table 1 t1:** Reports the positions of the aminoacids replaced in chimeric, truncated and double chimeric constructs.

Chimeras	Residues of TMEM16A Replaced by Equivalent Domain of TMEM16B (aa)
C-TERM	900–982
C-TERM 1	907–944
C-TERM 2	945–982
C-TERM 3	907–944 (945X)
C-TERM 4	907–931 (932X)
C-TERM 5	907–926 (928X)
C-TERM 6	907–922 (923X)
C-TERM 7	913–926 (928X)
C-TERM 8	917–926 (928X)
**Double Chimeras**	
N-TERM + C-TERM 1	1–341 and 907–944
TMD1-2 + C-TERM 1	347–380, 429–450 and 907–944
TMD3-4 + C-TERM 1	511–543, 556–583 and 907–944
TMD9-10 + C-TERM 1	779–816 and 879–944
LOOP1 + C-TERM 1	450–511 and 907–944

Further details may be provided on request.
