# Supplementary material for: Intermolecular Interactions in the TMEM16A Dimer Controlling Channel Activity
Source: Sci Rep. 2016 Dec 8;6:38788. doi: 10.1038/srep38788 (PMC5144075; doi:10.1038/srep38788)
Supplement: Supplementary Information [file srep38788-s1.pdf]

Supplementary Material

INTERMOLECULAR INTERACTIONS IN THE TMEM16A DIMER CONTROLLING CHANNEL ACTIVITY

Paolo Scudieri, Ilaria Musante, Ambra Gianotti, Oscar Moran, Luis J.V. Galietta

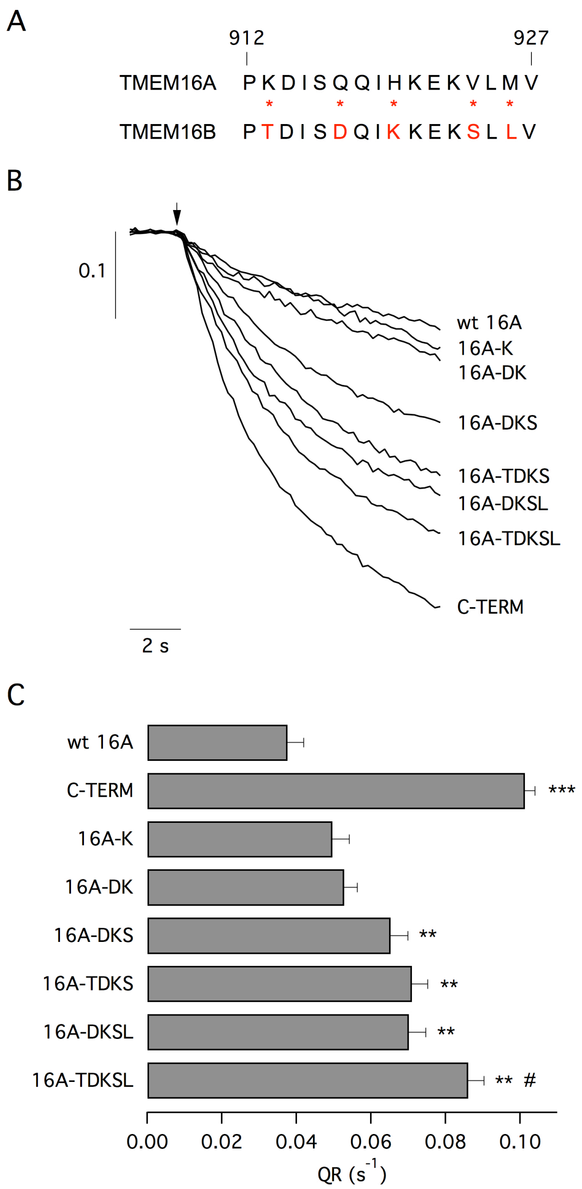

Supplementary Figure 1. Mutagenesis of the critical 912-927 region.

A. Alignment of TMEM16A-TMEM16B amino acid sequence in the region that affects basal activity. The residues that are different between the two proteins are evidenced.

B. Representative traces from HS-YFP assay showing activity (fluorescence decay) for the indicated TMEM16A proteins (mutant and wild type). The mutants are labeled with the symbol of the amino acid that was mutated. The arrow shows the time of addition of I^- solution (without ionomycin).

C. Activity for wild type and mutant TMEM16A measured with the HS-YFP assay. Data are reported as quenching rate (QR) of fluorescence decrease caused by I^- influx. **, $p < 0.01$ vs. wild type TMEM16A. #, $p < 0.05$ vs. triple and quadruple mutants (n = 12).

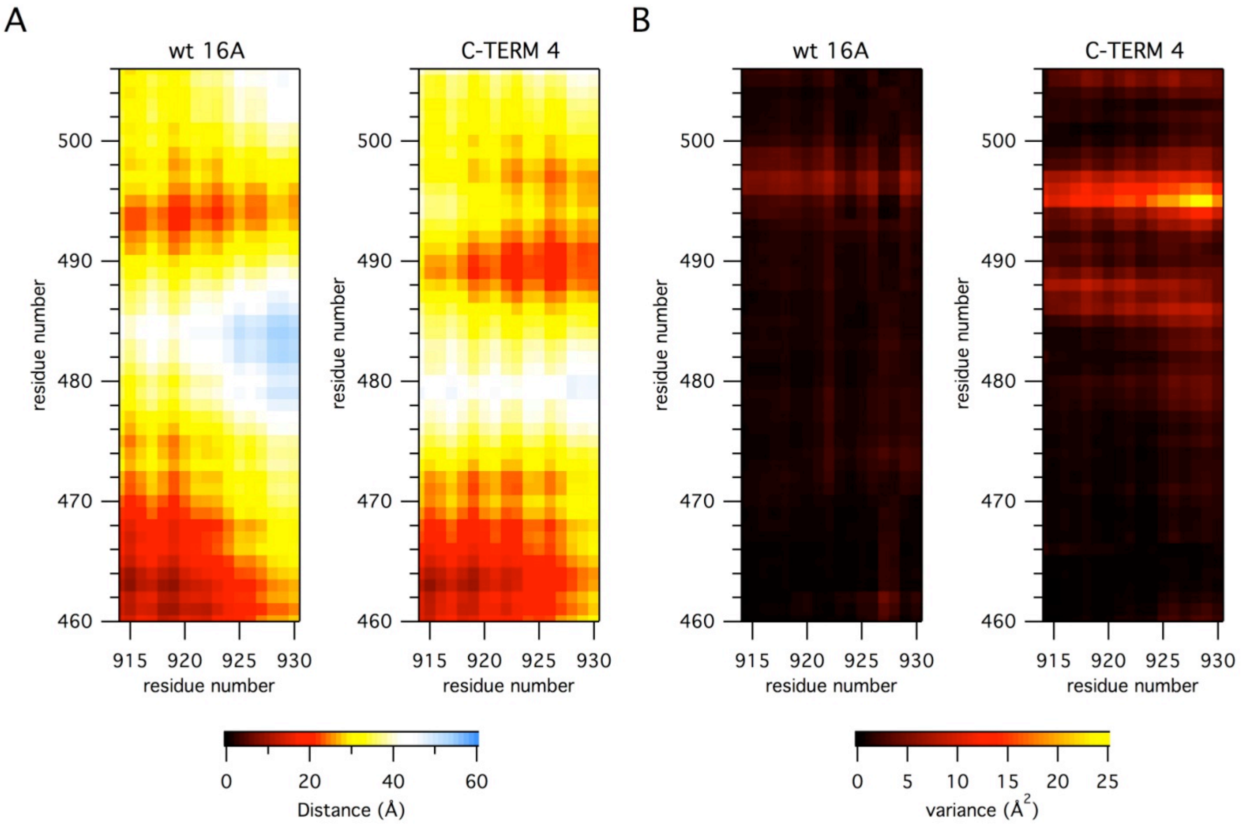

Supplementary Figure 2. Intrasubunit proximity between the C-terminus and the first intracellular loop.

A. Heatmaps showing the inter-residues average distance between the first α-helix of the C-terminus (x-axis) and first intracellular loop (y-axis) of the ipsilateral subunit in wild type TMEM16A and in C-TERM4 mutant.

B. Heatmaps showing the distance fluctuations (i.e. the variance of the inter-residues distance) between the first α-helix of the C-terminus (x-axis) and the first intracellular loop (y-axis) of the ipsilateral subunit in wild type TMEM16A and in C-TERM4 mutant. Data were obtained by molecular dynamics simulations as described in methods.

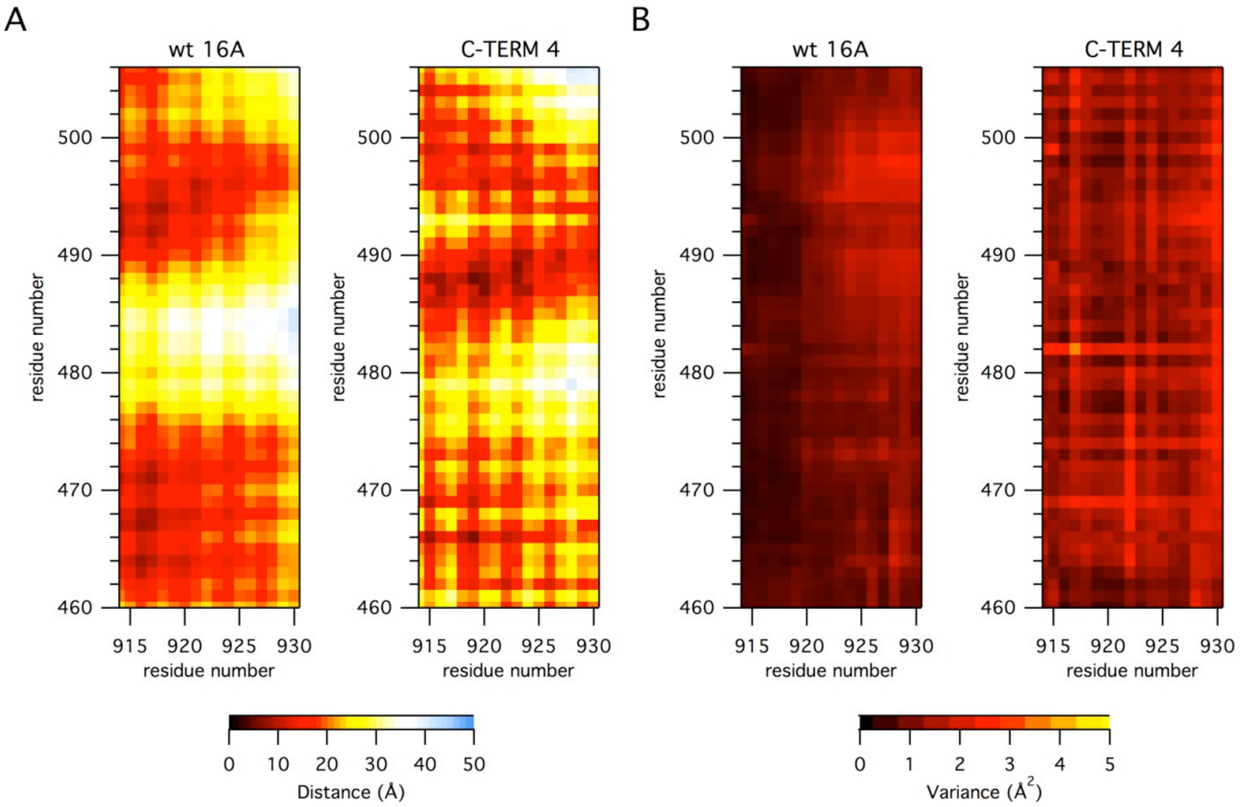

Supplementary Figure 3. Intersubunit proximity between C-terminus and first intracellular loop.

Heatmaps showing the inter-residues average distance (A) and the variance (B) between the first α-helix of the C-terminus (residues in the x-axis) and first intracellular loop (y-axis) of the contralateral subunit in wild type TMEM16A and in C-TERM4 mutant. Data were obtained by molecular dynamics simulations as described in methods.
